# News and Notes

**Published:** 1906-02

**Authors:** 


### *NEWS AND NOTES.*

DR. E. VAN GOIDTSNOVEN has recovered from his recent illness.

THE five medical schools of New York will this year take care of about 2,148 students.

SINCE January 1, 1905, there have been sixty thousand cases of typhoid fever in New York.

DR. DONALD B. FREDERICK, of Atlanta, and Miss May King, of Marshallville, Ga., were married December 27th.

PLANS for the new Army General Hospital, to be erected in Washington, D.C., have been accepted by Secretary Taft.

MRS. MILLICENT OLMSTEAD, of San Gabriel, Cal., has deeded $200,000 worth of property for the purpose of establishing a free hospital.

ACCORDING to the monthly *Homeopathic Review* of September 1 there are less than 300 homeopathic practitioners in the United Kingdom.

DR. FREDERICK G. BARFIELD, of Cuthbert, Ga., and Mrs. Helen Denham, of Jacksonville, Fla., were married November 29th.

NORWAY, it is claimed, forbids the newspapers to publish advertisements or in any other way to further the sale of any and all patent medicines.

An Austrian Society for the Repression of Quackery was formally constituted at a meeting held in the rooms of the Medical Society of Vienna recently.

---

Prof. William H. Welch, of Baltimore, has been elected a member of the Board of Trustees of the Carnegie Institution, in place of the late Secretary of State Hay.

---

The new sanatorium "Cragmount," for tuberculosis at Black Mountain, eleven miles above Asheville, is nearing completion and will be open about February 1st.

---

Middle Georgia Medical Association.—The physicians of this section met at Griffin December 8th, for the purpose of effecting a reorganization of the association.

---

In 1895 the degree of doctor of medicine was conferred on 6,011 medical students in the universities of Austria and Hungary. In 1904 only 561 similar degrees were conferred.

---

A bill has been introduced in the legislature giving physicians the same protection in the collection of fees for professional services in the last illnesses as are now afforded undertakers.

---

A warning has been issued by the United States Marine Hospital Service against lobsters, stating they are a cause of the spread of typhoid fever, which is prevalent now in many Eastern cities.

---

The President and entire State Board of Health of Louisiana have transmitted their resignations to Governor Blanchard. No appointments to fill the vacancies thus created have as yet been made.

FOUR HUNDRED AND THIRTY-EIGHT cases of diphtheria and fifty deaths were reported during the month of October to the division of contagious diseases of the Chicago Department of Health.

---

DR. GEO. H. NOBLE, of Atlanta was elected President of the Southern Surgical and Gynecological Association at the recent annual meeting in Louisville, Ky. The next meeting will be held in Baltimore.

---

DR. ARTHUR G. BEAZLEY, died at his home in Crawfordville, Ga., Christmas morning. Dr. Beazley was graduated from the Medical Department of the University of Virginia, in 1860, with first honors.

---

DR. NATHAN BOZEMAN, formerly of Macon, Ga., died at his home, in New York, of cerebral hemorrhage, December 16th, aged 80. His contributions to gynecological literature were numerous and valuable.

---

THE Chicago Medical Society has inaugurated the plan of having its members deliver popular lectures to the laity on subjects pertaining to medicine. The lectures are well attended, and the people take great interest in them.

---

DR. W. R. BRINCKERHOFF, of the Harvard Medical School, has been appointed pathologist in charge of the new hospital and laboratory at the government experiment station in the leper settlement on the Island of Molokai.

---

DR. B. W. TAYLOR, well known throughout South Carolina, died at his home in Columbia, December 27th, of pneumonia. Dr. Taylor was Colonel in the Confederate army on General Hampton's staff, and the chief surgeon in Charleston harbor at the fall of Fort Sumter.

---

SIR JOHN BURDON-SANDERSON, BART., M.D., one of England's most illustrious Physicians, died at his home in Oxford, November 23d, from broncho-pneumonia, after a prolonged illness.

Dr. Sanderson was 76 years of age and was once a regius professor of Medicine at Oxford, which position Dr. Osler now holds.

---

DR. ROBERT FLETCHER, associate librarian of the Army Medical Library, was entertained at a banquet by the local profession January 11th, and presented with a handsome loving-cup as a testimony of his valuable services in this library during the past thirty years. Dr. William W. Keen, Philadelphia, made the presentation speech.

---

THE physicians of Wilkes county met recently at Washington and perfected permanent organization on the standard plan. The new society has on its roll every practicing physician in the county. D. Thomas J. Willis was elected president; Dr. John J. Hill, vice-president, and Dr. A. W. Simpson, secretary and treasurer, all of Washington.

---

IT is reported that Prof. V. Czerny is to retire from the surgical clinic at Heidelberg in order to devote his energies exclusively to the Institute for Cancer Research, which was recently founded there mainly by his efforts and donations. Garrè, who recently took Mikulicz's vacant chair at Breslau, is suggested as Czerny's successor. Nearly $200,000 have been subscribed for the new cancer institute.

---

ALL hope of consolidating the Medical College of Virginia with the Medical Department of the University of Virginia has been given up. At a joint meeting of the committee just held it was found impracticable at this time for the amalgamation to be consummated. While the supreme end of union has not been reached, the long-existing friendship of the two institutions has been strengthened.

---

A NOTICE has been hung up in the Military Medical Academy, St. Petersburg, to the effect that unless all efforts are abandoned to make the academy a center of political action the committee will be obliged with regret to close the establishment. The notice appeals to the students to have sufficient reverence for the cause of education and the honor of the academy to make such a course uncalled for.—*Med. Age.*

---

A $2,000,000 TUBERCULOSIS hospital is planned for the poor of New York City, to be built on Staten Island. It will be constructed under the auspices of the Department of Charities; and will be separate from the undertaking of the Health Department, to build a sanitarium for incipient cases of consumption in Orange county, New York State. The Staten Island institution will be a hospital for cases in all stages of the disease.—*Med. Times.*

---

THE annual banquet of the Fulton County Medical Society was held in the Capital City Club December 30th, there being present about forty physicians. Owing to the absence of the President, Dr. E. Bates Block, the Vice-President, Dr. Claude Smith, presided. Among those who made after-dinner speeches were: Dr. A. W. Stirling, Dr. W. B. Emery, Dr. Dunbar Roy, Dr. W. S. Elkin, Dr. W. P. Nicholson, Dr. M. B. Hutchins, Dr. Michael Hoke and Dr. J. G. Earnest.

---

AT the annual meeting of the Chatham County, Ga., Medical Society, held at Savannah on Wednesday, December 13, 1905, the following officers were elected for the ensuing year: President, Dr.

John W. Daniel; vice-president, Dr. W. B. Crawford; secretary, Dr. H. W. Hesse; treasurer, Dr. W. E. Norton; censors, Dr. M. F. Dunn, Dr. M. H. Corbin, and Dr. J. C. Le Hardy. The programme committee appointed by the president consists of Dr. T. P. Waring, Dr. Martin Cooley, and Dr. H. W. Hesse.

---

DR. WILLIAM OSLER reached Baltimore from Canada, January 5th, and took up his quarters at the Johns Hopkins Hospital as the guest of Dr. Hurd. A reception was tendered Dr. Osler on the evening of January 6th, in the rotunda of the hospital, members of the faculty, the house physicians and medical students attending. January 23d he will deliver an address before the Maryland Association for the Relief and Prevention of Tuberculosis at Annapolis, in advocacy of a State Tuberculosis hospital.

---

A DINNER was given by the Floyd County, Ga., Medical Society, at Rome, on Thursday, December 21, 1905. The list of toasts prepared for the occasion, with the names of those who were to respond, was as follows: The Floyd County Medical Society, Dr. W. J. Shaw; Reminiscences of My Early Surgical Experience, Dr. H. H. Battey; What the Laity Expect of a Physician, Dr. J. N. Chenney; The Medical Fraternity, Dr. J. C. Watts; The Specialist Point of View, Dr. R. P. Cox; The Physician and Municipality, Dr. R. H. Wicker; The Importance of Organization, Dr. A. T. Calhoun; My First Difficult Case, Dr. J. Sewell; The Physician and the Microscope, Dr. W. L. Funkhouser; What the Physician Does for Charity, Dr. W. P. Harbin; The Public and Tuberculosis, Dr. L. P. Hammond.

---

IN two respects the medical profession deserves the grateful recognition and regard of all other callings in modern life. It has always insisted that the practice of medicine is a profession and not a trade. Trade is occupation for livelihood; profession is occupation for the service of the world. Trade is occupation for joy of the result; profession is occupation for joy in the process. Trade is occupation where anybody may enter; profession is occupation where only those who are prepared may enter. Trade is occupation taken up temporarily, until something better offers; profession is occupation with which one is identified for life. Trade makes one the rival of every other trade; profession makes one the co-operator with all his colleagues. Trade knows only the ethics of success; profession is bound by lasting ties of sacred honor.—*President Faunce of Brown University, addressing the Rhode Island Medical Society.*

---

THE CRAWFORD W. LONG MEMORIAL ASSOCIATION.—The executive committee of the association, appointed by the Georgia Medical Association to attend to the matter of raising funds for a suitable monument for Dr. Crawford W. Long as the discoverer of anesthesia, to be placed in the Hall of Fame at Washington, held a meeting in the capitol at Atlanta on Tuesday, January 2d, Dr. Willis F. Westmoreland, chairman, presiding. It is the purpose of the committee to raise a substantial fund and then ask the Legislature for an appropriation. The members of the memorial association are as follows: Dr. Willis F. Westmoreland, of Atlanta, chairman; Dr. W. W. Owens, of Savannah; Dr. Alexander Mack, of Decatur; Dr. J. D. Chason, of Bainbridge; Dr. F. M. Ridley, of LaGrange; Dr. Floyd McRae, of Atlanta; Dr. H. J. Williams, of Macon; Dr. R. M. Harbin, of Rome; Dr. S. C. Benedict, of Athens; Dr. L. G. Hardman, of Commerce; Dr. J. B. Morgan, of Augusta, and Dr. Charles Hicks, of Dublin.

